# Roles of CREB in the regulation of FMRP by group I metabotropic glutamate receptors in cingulate cortex

**DOI:** 10.1186/1756-6606-5-27

**Published:** 2012-08-06

**Authors:** Hansen Wang, Yoshikazu Morishita, Daiki Miura, Jose R Naranjo, Satoshi Kida, Min Zhuo

**Affiliations:** 1 Department of Physiology, Faculty of Medicine, University of Toronto, 1 King’s College Circle, Toronto, ON, M5S 1A8, Canada; 2 Department of Bioscience, Faculty of Applied Bioscience, Tokyo University of Agriculture, Tokyo, 156-8502, Japan; 3 Centro Nacional de Biotecnologia, Consejo Superior de Investigaciones Científicas and Centro Investigaciones Biomedicas En Red-NEuroDegenerativas, E-28049, Madrid, Spain; 4 Center for Neuron and Disease, Frontier Institute of Science and Technology, Xi’an Jiaotong University, Xi’an, China; 5 Department of Physiology, University of Toronto, Faculty of Medicine, 1 King’s College Circle, Toronto, ON, M5S 1A8, Canada

**Keywords:** CREB, FMRP, Group I mGluRs, Gene expression, Cingulate cortex, Fragile X syndrome

## Abstract

**Background:**

Fragile X syndrome is caused by lack of fragile X mental retardation protein (FMRP) due to silencing of the FMR1 gene. The metabotropic glutamate receptors (mGluRs) in the central nervous system contribute to higher brain functions including learning/memory, mental disorders and persistent pain. The transcription factor cyclic AMP-responsive element binding protein (CREB) is involved in important neuronal functions, such as synaptic plasticity and neuronal survival. Our recent study has shown that stimulation of Group I mGluRs upregulated FMRP and activated CREB in anterior cingulate cortex (ACC), a key region for brain cognitive and executive functions, suggesting that activation of Group I mGluRs may upregulate FMRP through CREB signaling pathway.

**Results:**

In this study, we demonstrate that CREB contributes to the regulation of FMRP by Group I mGluRs. In ACC neurons of adult mice overexpressing dominant active CREB mutant, the upregulation of FMRP by stimulating Group I mGluR is enhanced compared to wild-type mice. However, the regulation of FMRP by Group I mGluRs is not altered by overexpression of Ca^2+^-insensitive mutant form of downstream regulatory element antagonist modulator (DREAM), a transcriptional repressor involved in synaptic transmission and plasticity.

**Conclusion:**

Our study has provided further evidence for CREB involvement in regulation of FMRP by Group I mGluRs in ACC neurons, and may help to elucidate the pathogenesis of fragile X syndrome.

## Background

Fragile X syndrome, the most common cause of inherited mental retardation and autism spectrum disorders, is caused by mutations of the *FMR1* gene that encodes the fragile X mental retardation protein (FMRP) [1-9]. FMRP, an mRNA binding protein, is involved in activity-dependent synaptic plasticity through regulation of local protein synthesis at synapses [2,7,9-16]. It normally functions as a repressor of translation of specific mRNAs [10,15,17-19]. The abnormal functions of Group I mGluR-dependent synaptic plasticity have been observed in hippocampus of *Fmr1* knockout (KO) mice [16,17,20-23]. It is believed that the protein synthesis downstream of Group I mGluRs are exaggerated due to the lack of FMRP in fragile X syndrome [8,17,21,24].

The anterior cingulate cortex (ACC) is important for cognitive learning, fear memory and persistent pain [25-31]. Previous studies have shown that trace fear memory is impaired in *Fmr1* KO mice, accompanied by alterations in synaptic plasticity in ACC, suggesting that the dysfunction of ACC due to lack of FMRP may be responsible for certain types of mental disorders in fragile X syndrome [27,32]. The mGluRs in ACC contribute to activity-dependent synaptic plasticity and behavioral fear memory [33,34]. The regulation of FMRP by mGluRs has been mostly studied in hippocampal neurons [11,17,21,35,36]. Our recent study has found that activation of Group I mGluRs regulates the expression of FMRP in ACC neurons and activates cyclic AMP-responsive element binding protein (CREB) [37,38], a transcriptional factor which plays many functional roles in central nervous system, such as neuronal survival, synaptic plasticity, learning and memory [39-45]. These findings indicate possible roles of CREB in linking mGluRs to FMRP in ACC. Loss of this signaling pathway may contribute to the pathogenesis of fragile X syndrome.

In the present study, we have demonstrated that CREB is involved in the regulation of FMRP by Group I mGluRs. In cingulate cortex from transgenic mice overexpressing dominant active CREB (Y134F) mutant which displays a higher affinity with cAMP dependent kinase (PKA) compared to wild-type (WT) CREB [46,47], we found the upregulation of FMRP by stimulating Group I mGluR was enhanced compared to that of WT mice. By contrast, the regulation of FMRP by Group I mGluRs was not affected by overexpression of Ca^2+^ insentive mutant form of downstream regulatory element antagonist modulator (DREAM), a transcriptional repressor involved in synaptic plasticity, learning and memory [48-50]. We propose that CREB is the key transcription factor in regulation of FMRP by Group I mGluRs in ACC neurons.

## Results

### Overexpression of dominant active CREB enhances the regulation of FMRP by group I mGluRs in the ACC neurons

Phosphorylated CREB (pCREB) binds to cAMP response element (CRE) site in gene promoters and activates gene transcription [41,42,45,51,52]. It has been reported that the *FMR1* gene promoter contains the CRE site [53,54]. Our recent study had found that (RS)-3, 5-Dihydroxyphenylglycine ((RS)-3, 5-DHPG) treatment could upregulate FMRP and increase the pCREB levels in ACC slices, suggesting that the regulation of FMRP by Group I mGluRs in ACC neurons likely occurs through CREB activation [37,38].

**Figure 1:**
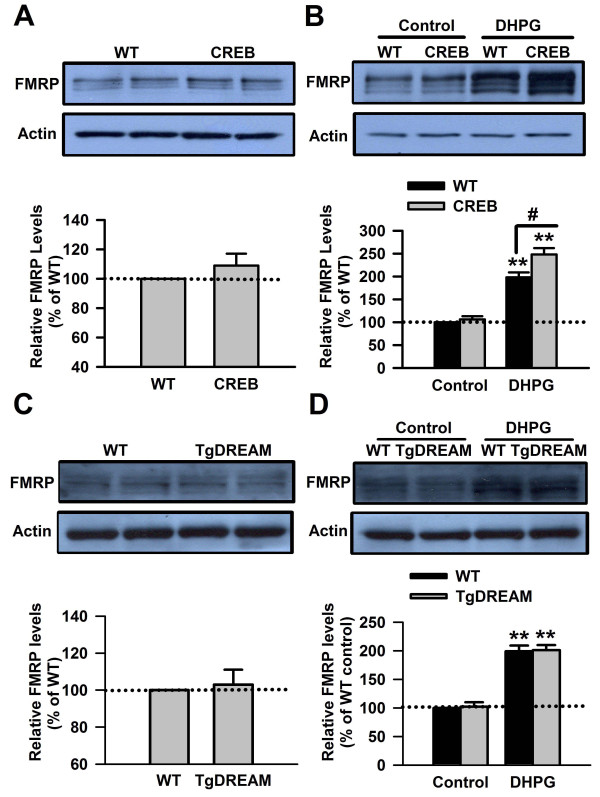
**Upregulation of FMRP by Group I mGluRs was enhanced in ACC from CREB mutant mice, whereas it was not affected in transgenic mice overexpressing a Ca**^**2+**^**-insensitive DREAM mutant (TgDREAM). A**, The basal levels of FMRP in ACC slices of CREB mutant mice were not affected. **B**, The increase of FMRP after treatment with Group I mGluR agonist DHPG (100 μM) for 30 min, was enhanced in ACC slices from CREB mutant mice, as compared to wild-type (WT) mice. Representative Western blot (top) and quantification data (bottom) of FMRP are shown for the corresponding treatments. **C**, The basal levels of FMRP in ACC slices of TgDREAM mice were not affected. **D**, The increase of FMRP after treatment with Group I mGluR agonist DHPG (100 μM) for 30 min, was not changed in ACC slices from TgDREAM mutant mice, as compared to wild-type (WT) mice. Representative Western blot (top) and quantification data (bottom) of FMRP are shown for the corresponding treatments. Data were normalized by WT control values. ** *P* < 0.01, compared to control mice; # *P* < 0.05, compared to WT DHPG treatment. *n* = 5 mice for each group.

Overexpression of dominant active CREB mutant in the forebrain could positively regulate memory consolidation and enhance memory performance by upregulating the expression of Brain derived neurotrophic factor (BDNF) [47], which is well known as a CREB target gene [40,42,55]. To further investigate whether CREB is involved in the upregulation of FMRP caused by stimulating Group I mGluRs, we then tested the expression of FMRP induced by the Group I mGluR agonist DHPG (100 μM, 30 min) treatment in ACC slices from mice overexpressing CREB. By Western blot, we found that there was no difference in the basal levels of FMRP in ACC slices between WT and CREB overexpression mice (*P* > 0.05, compared with WT mice, *n* = 5, Figure 1A). DHPG treatment could increase expression of FMRP in ACC slices; the increase of FMRP was further enhanced in ACC slices from mice overexpressing CREB compared to WT mice (198 ± 11% and 248 ± 14% of the WT control levels for WT and CREB overexpression mice, respectively. In two-way ANOVA analysis, for genotype, *F* = 13.39, *P* < 0.01; for treatment, *F* = 254.87, *P* < 0.01; genotype X treatment, *F* = 8.26, *P* < 0.05; *n* = 5 for each group, Figure 1B). The data indicates that overexpression of CREB can enhance the upregulation of FMRP induced by Group I mGluR activation. It provides further evidence that CREB is involved in the regulation of FMRP by Group I mGluRs in ACC neurons. 

### Overexpression of Ca^2+^-insensitive DREAM does not affect the regulation of FMRP by group I mGluRs in the ACC neurons

Since transcriptional repressor DREAM interacts with CREB in a Ca^2+^ dependent manner and prevents the recruitment of CREB-binding protein (CBP) blocking CRE-dependent gene transcription [48,56], we next checked whether DREAM might be involved in the regulation of FMRP by Group I mGluRs through CREB signaling pathway. To explore the role of DREAM in the upregulation of FMRP by stimulating Group I mGluRs, we have taken the advantage of transgenic mice overexpressing a Ca^2+^-insensitive DREAM mutant (TgDREAM) [49,50]. The TgDREAM mice could develop normally and did not exhibit any abnormalities in brain structures. However, overexpression of mutant DREAM impaired NMDA receptor-mediated synaptic plasticity and contextual fear memory [50].

We next tested the effect of DHPG (100 μM, 30 min) treatment in ACC slices from TgDREAM mice. Importantly, no difference in the basal levels of FMRP in ACC slices was observed between WT and TgDREAM mice (P > 0.05, compared with WT mice, n = 5, Figure 1C). Furthermore, the increase of FMRP after DHPG treatment was not affected in ACC slices from TgDREAM mice compared to WT mice (199 ± 10% and 201 ± 9% of the WT control levels for WT and TgDREAM mice, respectively. In two-way ANOVA analysis, for genotype, *F* = 0.10, *P* = 0.75; for treatment, *F* = 249.81, *P* < 0.01; for genotype X treatment, *F* = 0.001, *P* = 1.00; *n* = 5 for each group, Figure 1D). The data indicate that overexpression of Ca^2+^-insensitive mutant form of DREAM does not affect the upregulation of FMRP induced by Group I mGluR activation, suggesting that DREAM might not be involved in the CREB-dependent regulation of FMRP by Group I mGluRs in ACC neurons.

### Putative CREs in the *FMR1* promoter

**Figure 2:**
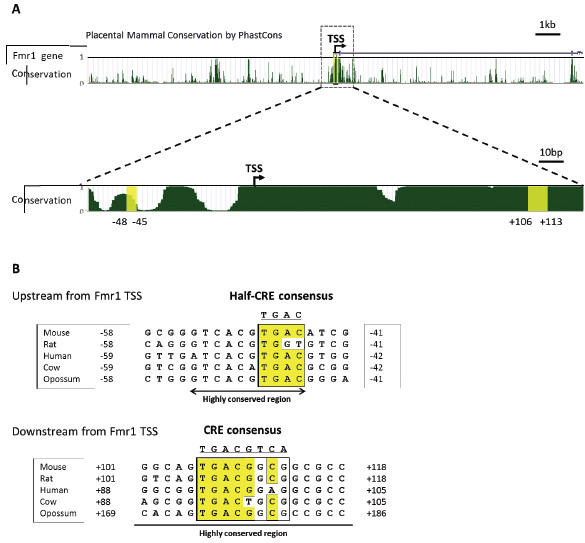
**Putative CREs in the *FMR1 *promoter. A**, Conserved regions among multiple placental mammalian species were identified by UCSC Genome browser. Two putative CREs are indicated with yellow. TSS; transcription start site. **B**, Two putative CREs are highly conserved across species (mouse, rat, human, cow, opossum). Conserved CRE sequences are highlighted in yellow.

To identify conserved sequences, 20 kb of mouse genomic sequence including the *FMR1* transcription start site (TSS) was aligned among multiple mammalian species using the UCSC Genome browser (Figure 2). Sequences of multiple mammalian species were then scanned for matches to the consensus sequence of CRE (TGACGTCA). Two putative CREs (upstream CRE, -48 ~ −45; downstream CRE, +106 ~ +113) were found in the highly conserved regions across multiple mammals (Figure 2v). The upstream putative CRE has been reported as a potential CRE in human *FMR1* promoter [53,54]. Comparisons of putative CRE sequences among mammalian species are shown in Figure 2B. These data support our finding that the *FMR1* is a target gene of CREB. 

## Discussion

Our previous studies have shown that FMRP is required for the physiological function of ACC [25,27,57], the mGluRs in ACC may contribute to the activity-dependent synaptic plasticity and fear memory [33,34]. Recently, we have provided the direct biochemical evidence that activation of Group I mGluRs upregulates FMRP in ACC neurons of adult mice; the upregulation of FMRP by Group I mGluRs occurs at the transcriptional level, stimulation of Group I mGluRs induced the phosphorylation of CREB in ACC neurons [37,38]. In this study, we provided further evidence that CREB contributes to the upregulation of FMRP induced by stimulating Group I mGluRs and may act as a key signaling molecule linking Group I mGluRs and FMRP in cingulate cortex.

CREB is a transcriptional factor that plays important roles in synaptic plasticity [40-45,52]. The activity of CREB is regulated by its phosphorylation; pCREB binds to the CRE site within the gene and activates the gene transcription [40,42,45,51,52]. Previous and our current studies have shown that there is the CRE site in *FMR1* promoter, and implicated CREB in the regulation of the *FMR1* gene transcription in neural cells (Figure 2) [53,54]. Our recent studies found that the Group I mGluR activation upregulates FMRP at the transcriptional level in ACC neurons; the upregulation of FMRP is accompanied by the phosphorylation of CREB (Ser133); Ca^2+^-stimulated adenylyl cyclase 1 (AC1), PKA and Ca^2+^/Calmodulin-dependent protein kinase IV (CaMKIV) contribute to regulation of FMRP by Group I mGluR probably through CREB activation [37,38] (see Table 1). These findings supported that CREB acts as a transcriptional factor for Group I mGluR-dependent upregulation of FMRP in the ACC neurons. 

**Table 1:** Studies on signaling pathway of CREB activation by Group I mGluRs in cingulate cortex

**Signaling molecules**	**Manipulations**	**Effects on CREB phosphorylation induced by DHPG**	**References**
AC1	*AC1* knockout	Reduced	38
PKA	PKA inhibitor	Reduced	38
CaMKIV	*CaMKIV* knockout	Reduced	38
	CaMK inhibitor	Reduced	37
	*CaMKIV* over expression	Enhanced	37

**Figure 3:**
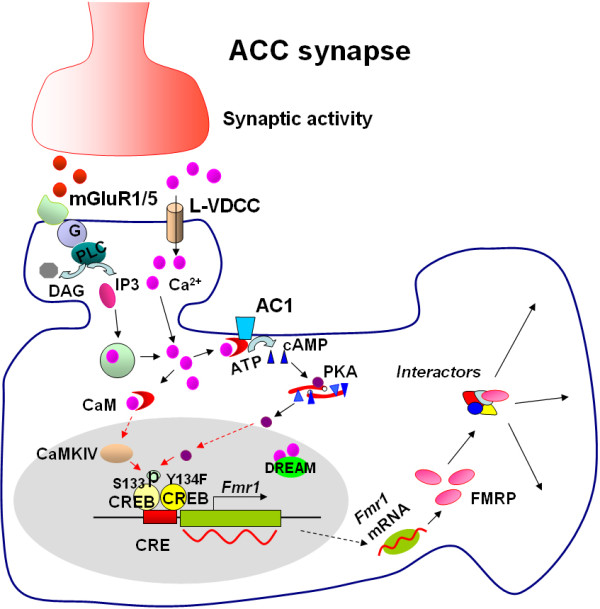
**The signaling pathway for CREB in the regulation of *FMRP *by Group I mGluRs in ACC neurons.** Stimulation of mGluR1/5 triggers the Ca^2+^ release from intracellular calcium stores by IP3 and Ca^2+^ influx from L-VDCCs through membrane depolarization. The increase of Ca^2+^ leads to activation of Ca^2+^-calmodulin (CaM) dependent pathways, including Ca^2+^ and CaM stimulated AC1-cAMP dependent protein kinase (PKA) and CaMKIV. PKA and CaMKIV then phosphorylates CREB. Phosphorylated CREB (pCREB) initiates the CREB-dependent transcription of *Fmr1* gene and upregulates FMRP in the cytoplasm. The mutant CREB (Y134F) contributes to transcription of *Fmr1* gene, whereas DREAM may not be involved in *Fmr1* gene expression. FMRP may interact with its interactors and modulate neuronal functions in ACC.

In this study, we have shown that the upregulation of FMRP induced by Group I mGluR agonist DHPG DHPG is enhanced in ACC slices from mice overexpressing dominant active CREB (Y134F) mutant. This finding further supports that CREB is critical for the regulation of FMRP by Group I mGluRs in ACC neurons. We also found that overexpression of dominant active CREB mutant does not affect the basal levels of FMRP, although it enhanced the upregulation of FMRP by stimulating Group I mGluRs in ACC slices. These results may reflect less synaptic activity at baseline condition, or suggest that CREB, which can be shared by many different signaling pathways, may specifically contribute to the upregulation of FMRP by stimulating Group I mGluRs (see Figure 3 for the model). It is possible that long term expression of dominant active CREB in the mice may cause some developmental or secondary changes in ACC of transgenic mice. However, we think that the effect of CREB mutant on regulation of FMRP by Group I mGluRs cannot be simply attributed to developmental or secondary changes in ACC since the roles of CREB have been further supported by other genetic and pharmacological evidence from our previous studies [37,38]. 

DREAM, a multifunctional Ca^2+^-binding protein, contributes to synaptic plasticity, and behavioral learning and memory. As a transcriptional repressor, it can affect CRE-dependent gene transcription by preventing the recruitment of CBP by pCREB [48,49,56]. In this study, we found the upregulation of FMRP by stimulating Group I mGluRs was not affected in ACC slices from mice overexpressing Ca^2+^-insensitive mutant form of DREAM. The data indicates that overexpression of this mutant form of DREAM does not affect basal expression or CREB-dependent FMRP induction by Group I mGluRs. Since the overexpression of TgDREAM has been associated with the repression of different target genes [49,58,59], these results suggest that DREAM might not be involved in the regulation of the FMRP in ACC neurons.

## Conclusion

We have demonstrated that CREB is critical for regulation of FMRP by Group I mGluRs in ACC neurons by using genetic approaches. Our study has provided further evidence that CREB is involved in regulation of FMRP by Group I mGluRs in cingulate cortex, and may help to further elucidate the molecular and cellular mechanisms underlying fragile X syndrome.

## Materials and methods

### Animals

Adult male C57Bl/6 mice were used in most of experiments. The transgenic mice overexpressing dominant active mutant CREB (Y134F) or Ca^2+^ insentive DREAM were generated and maintained as reported previously [47,50]. All mice were housed under a 12:12 light cycle with food and water provided ad libitum. All mouse protocols are in accordance with NIH guidelines and approved by the Animal Care and Use Committee of University of Toronto.

### Drugs and antibodies

(RS)-3, 5-DHPG was purchased from Tocris Bioscience (Ellisville, MO). phosphatase inhibitor cocktail 1 and 2 were purchased from Sigma-Aldrich (St. Louis, MO). The anti-FMRP antibody, horseradish peroxidase-linked goat anti-mouse IgG and goat anti-rabbit IgG for Western blot were purchased from Chemicon International (Temecula, CA). The anti-phospho-threonine antibody, anti-CREB antibody and anti-phosph CREB antibody were purchased from Cell Signaling Technology (Danvers, MA). The anti-actin antibody was from Sigma-Aldrich (St. Louis, MO).

### Brain slice preparations

Mice were anesthetized with 2% halothane and brain slices (300 μm) containing ACC were cut at 4°C using a Vibratome, in oxygenated artificial cerebrospinal fluid [ACSF; containing the following (in mM): 124 NaCl, 4 KCl, 26 NaHCO_3_, 2.0 CaCl2, 1.0 MgSO_4_, 1.0 NaH_2_PO_4_, 10 D-glucose, pH 7.4]. The slices were slowly brought to final temperature of 30°C in ACSF gassed with 95% O_2_/5% CO_2_ and incubated for at least 1 hour before experiments. Slices then were exposed to different compounds of interest for the indicated times and snap frozen over dry ice. For biochemical experiments, the ACC regions were microdissected and sonicated in ice-cold homogenization buffer containing phosphatase and protease inhibitors.

### Western blot analysis

Western blot was conducted as previously described [25,38]. The brain tissues were dissected and homogenized in lysis buffer containing 10 mM Tris–HCl (pH 7.4), 2 mM EDTA, 1% SDS, 1X protease inhibitor cocktail, and 1X phosphatase inhibitor cocktail 1 and 2. Protein concentration was measured by Bradford protein assay (Bio-Rad, Hercules, CA). Electrophoresis of equal amounts of total protein was performed on NuPAGE 4-12% Bis-Tris Gels (Invitrogen, Carlsbad, CA). Separated proteins were transferred to polyvinylidene fluoride membranes (Pall Corporation, East Hills, NY) at 4°C for analysis. Membranes were probed with 1:3000 dilution of anti-FMRP, or 1:1000 dilution of anti-phospho-CREB (Ser133) and anti-CREB antibodies. The membranes were incubated in the appropriate horseradish peroxidase-coupled secondary antibody diluted 1:3000 for 2 h followed by enhanced chemiluminescence (ECL) detection of the proteins with Western Lightning Plus-ECL (PerkinElmer Life and Analytical Science Inc., Waltham, MA) according to the manufacturer’s instructions. To verify equal loading, membranes were also probed with 1:3000 dilution of anti-actin antibody. The density of immunoblots was measured using NIH ImageJ program.

### Data analysis

All data were presented as the mean ± S.E.M. Statistical comparisons were performed by paired *t*-test or two-way ANOVA. In all cases, *P* < 0.05 is considered statistically significant.

## Abbreviations

FMRP: Fragile X mental retardation protein; mGluRs: Metabotropic glutamate receptors; CaMKIV: Ca^2+^/calmodulin-dependent protein kinase IV; AC1: Adenylyl cyclase 1; ACC: Anterior cingulate cortex; DHPG: (RS)-3: 5-Dihydroxyphenylglycine; CREB: Cyclic AMP-responsive element binding protein; pCREB: Phosphorylated CREB; DREAM: Downstream regulatory element antagonist modulator; PKA: cAMP dependent kinase; WT: Wild-type.

## Competing interests

The authors declare that they have no competing interests.

## Authors’ contributions

Hansen Wang and Min Zhuo designed the study and wrote the manuscript. Hansen Wang performed the experiments. Yoshikazu Morishita, Daiki Miura and Satoshi Kida provided the CREB mutant mice and analyzed CRE sequences in the *FMR1* promoter. Jose R Naranjo provided the DREAM mutant mice. Min Zhuo supervised the study. All authors read and approved the final manuscript.
